# CXCL5 signaling is a shared pathway of neuroinflammation and blood–brain barrier injury contributing to white matter injury in the immature brain

**DOI:** 10.1186/s12974-015-0474-6

**Published:** 2016-01-06

**Authors:** Lin-Yu Wang, Yi-Fang Tu, Yung-Chieh Lin, Chao-Ching Huang

**Affiliations:** Department of Pediatrics, Chi Mei Medical Center, Tainan, 710 Taiwan; Department of Childhood Education and Nursery, Chia Nan University of Pharmacy and Science, Tainan, Taiwan; Department of Pediatrics, National Cheng Kung University Hospital, College of Medicine, National Cheng Kung University, Tainan, 704 Taiwan; Institute of Clinical Medicine, College of Medicine, National Cheng Kung University, Tainan, 704 Taiwan; Department of Pediatrics, College of Medicine, Taipei Medical University, Taipei, 110 Taiwan; Department of Pediatrics, Wan-Fang Hospital, Taipei Medical University, Taipei, 110 Taiwan

**Keywords:** CXCL5, CXCR2, Neuroinflammation, Blood–brain barrier, Lipopolysaccharide, Hypoxic ischemia, White matter injury, Immature brain, Preterm infant

## Abstract

**Background:**

In very preterm infants, white matter injury is a prominent brain injury, and hypoxic ischemia (HI) and infection are the two primary pathogenic factors of this injury. Microglia and microvascular endothelial cells closely interact; therefore, a common signaling pathway may cause neuroinflammation and blood–brain barrier (BBB) damage after injury to the immature brain. CXC chemokine ligand 5 (CXCL5) is produced in inflammatory and endothelial cells by various organs in response to insults. CXCL5 levels markedly increased in the amniotic cavity in response to intrauterine infection and preterm birth in clinical studies. The objective of this study is to determine whether CXCL5 signaling is a shared pathway of neuroinflammation and BBB injury that contributes to white matter injury in the immature brain.

**Methods:**

Postpartum day 2 (P2) rat pups received lipopolysaccharide (LPS) followed by 90-min HI. Immunohistochemical analyses were performed to determine microglial activation, neutrophil infiltration, BBB damage, and myelin basic protein and glial fibrillary acidic protein expression. Immunofluorescence experiments were performed to determine the cellular distribution of CXCL5. Pharmacological tests were performed to inhibit or enhance CXCL5 activity.

**Results:**

On P2, predominant increases in microglial activation and BBB damage were observed 24 h after LPS-sensitized HI induction, and white matter injury (decreased myelination and increased astrogliosis) was observed on P12 compared with controls. Immunohistochemical analyses revealed increased CXCL5 expression in the white matter 6 and 24 h after insult. Immunofluorescence experiments revealed upregulated CXCL5 expression in the activated microglia and endothelial cells 24 h after insult. CXCL5 inhibition by SB225002, a selective nonpeptide inhibitor of CXCR2, significantly attenuated microglial activation and BBB damage, increased myelination, and reduced astrogliosis in the white matter after LPS-sensitized HI. In addition, CXCL5-sensitized HI or CXCL5 alone significantly induced BBB damage and white matter injury in association with different neuroinflammation mechanisms. CXCL5-sensitized HI-induced microglial activation and neutrophil infiltration, whereas CXCL5 alone predominately caused neutrophil infiltration.

**Conclusions:**

CXCL5 is a potential biomarker for white matter injury in preterm infants. Pharmacological blockade of CXCL5 signaling that attenuates dysregulated neuroinflammation can be used a therapeutic strategy against white matter injury in the immature brain.

**Electronic supplementary material:**

The online version of this article (doi:10.1186/s12974-015-0474-6) contains supplementary material, which is available to authorized users.

## Background

Cytokines from monocytes and macrophages initiate inflammation by inducing chemokines released from inflammatory and endothelial cells [1, 2]. Chemokines, the principal factors in leukocyte migration during inflammation, can propagate the process through the recruitment and activation of additional cellular inflammatory mediators, such as macrophages and neutrophils. CXC chemokine ligand 5 (CXCL5) is a small cytokine belonging to the CXC chemokine family that is also known as epithelial neutrophil-activating peptide-78. CXCL5 is expressed in many different cells, including monocytes, endothelial cells, and alveolar epithelial type II cells [3, 4]. It is expressed in various organs, such as the brain, during endotoxemia [5]. CXCL5 is also produced by immune and vascular endothelial cells in response to proinflammatory cytokines through NF-kB activation [3, 6].

Cerebral white matter injury is a prominent brain injury and a leading cause of cerebral palsy in preterm infants [7]. The neuropathological hallmark of white matter injury in preterm infants is severe neuroinflammation and focal and diffuse white matter lesions along with astrocytosis and hypomyelination at early and late stages, respectively [7]. During insults, inflammatory cells may exacerbate white matter injury through the production of proinflammatory cytokines [7, 8]. The damaged microvessels may recruit activated leukocytes at the injured white matter through the disrupted blood–brain barrier (BBB), resulting in sustained neuroinflammation, which contributes to the exacerbation of white matter injury through the prolonged production of inflammatory cytokines or chemokines [9, 10]. Leukocytes and vascular endothelial cells may closely interact; therefore, a common mechanism associated with neuroinflammation and BBB damage and subsequent white matter injury may exist in the immature brain.

CXCL5 levels markedly increased in the amniotic cavity in response to intrauterine infection and preterm birth and in the serum of newborns with hypoxic ischemia (HI) encephalopathy in clinical studies [11, 12]. In addition, elevated CXCL5 levels were observed in the gastric fluids of preterm infants at birth, who subsequently developed severe bronchopulmonary dysplasia [13], and in the surgically resected intestinal samples of preterm infants with necrotizing enterocolitis [14]. However, whether CXCL5 plays a key role in white matter injury of the immature brain remains unknown.

The target cells of damage in white matter injury during the window of vulnerability in preterm infants are O4-positive oligodendrocyte progenitors [15]. The predominance of premyelinating oligodendrocytes in postpartum day 2 (P2) rat pups coincides with the high-risk period of white matter injury in very preterm infants [16]. HI and inflammation are the two primary risk factors of white matter injury and cerebral palsy in very preterm infants [7, 10]. The potentiating effect of infection on HI-induced injury in the white matter of the immature brain has been reported in several studies [17, 18]. Our previous study on P2 rat pups demonstrated that low-dose lipopolysaccharide (LPS) or 90-min HI alone caused no considerable injury in the cortex or white matter, whereas a combination of the two caused selective white matter injury [18]. These findings suggest that LPS sensitizes HI and selectively causes white matter injury in the immature brain. CXCL5, the chemokine that attracts and activates leukocytes, has been proposed as a potential therapeutic target in several inflammatory diseases [19]. Thus, using pharmacologic approaches in the P2 rat pups (brain maturation status equivalent to human gestation <28 weeks), we hypothesized that CXCL5 signaling is the shared pathway associated with neuroinflammation, BBB damage, and subsequent white matter injury in the immature brain.

## Methods

This study was approved by the Animal Care Committee of National Cheng Kung University. Technicians and investigators blinded to the grouping performed the experiments and quantitative measurements, respectively.

### Rat pup model of LPS-sensitized HI white matter injury

In this study, 10–12 Sprague–Dawley rat pups per dam were housed with a 12-h light–dark cycle and cared for according to the National Institutes of Health guidelines. The P2 Sprague–Dawley rat pups received intraperitoneal 0.05 mg/kg LPS (*Escherichia coli* 0111:B4; Sigma-Aldrich) or normal saline (NS) injection 3 h before HI. To avoid LPS-induced body temperature changes, the pups were returned to their dams after LPS or NS administration and housed in an incubator to maintain the body temperature at 33–34 °C before HI. Subsequently, HI was induced by ligating the right common carotid artery and inducing hypoxia. The right common carotid artery was permanently ligated under 2.5 % halothane anesthesia. After surgery, the pups were returned to an incubator for 1-h recovery. Subsequently, the rats were placed in airtight 500-mL containers partially submerged in 36 °C water baths. The containers were humidified with 6.5 % oxygen at a flow rate of 3 L/min for 90 min [18]. Following hypoxia, the pups were returned to their dam and randomly assigned to three groups: control (NS without HI, *N* = 12), NS + HI (NS injection 3 h before HI, *N* = 12), and LPS + HI (LPS injection 3 h before HI, *N* = 12).

### Pharmacological inhibition of CXCR2 in LPS-sensitized HI

SB225002, a selective nonpeptide inhibitor of CXCR2, the receptor of CXCL5, was used for inhibiting CXCL5 activity [20]. The pups received intraperitoneal injections of SB225002 (1 or 3 mg/kg, diluted in NS containing 0.33 % Tween 80, Cayman Chemical) or vehicle (NS solution containing 0.33 % Tween 80) 30 min before LPS administration and immediately after HI. The pups were randomly assigned to four groups: control (pups unexposed to LPS or HI, *N* = 14), vehicle (NS injections 30 min before LPS administration and immediately after HI, *N* = 18), and SB-1 (1 mg/kg, *N* = 14) and SB-3 (3 mg/kg, *N* = 18) (SB225002 injections 30 min before LPS administration and immediately after HI). The SB225002 dose used in this study was modified from that used by Bento et al. [20].

### Intracerebroventricular CXCL5 infusion

The P2 pups were anesthetized using 2.5 % halothane and intracerebroventricularly infused with recombinant rat CXCL5 (25 μg of CXCL5 dissolved in 50 μL of NS, R&D Systems) or NS into the right cerebral hemisphere by using a 30-gauge needle on a 10-μL Hamilton syringe. The location of each injection in relation to the bregma was 2.0 mm posterior to, 1.5 mm lateral to, and 2.0 mm beneath the skull surface [21]. Each pup received 2 μL (1 μg) or 4 μL (2 μg) of CXCL5.

Two separate CXCL5 injection-related experiments were performed. In the HI experiment, CXCL5 or the vehicle was infused 3 h before HI. Accordingly, five groups were formed: control (without LPS or HI, *N* = 12), LPS + HI (LPS 3 h before HI, *N* = 12), NS + HI (NS before HI, *N* = 12), CXCL5-1 + HI (1-μg CXCL5 before HI, *N* = 12), and CXCL5-2 + HI (2-μg CXCL5 before HI, *N* = 12). In the non-HI experiment, two groups were formed: vehicle (NS, *N* = 12) and CXCL5 (2 μg, *N* = 12).

### Immunohistochemical analyses

The pups were sacrificed, and cryo- and paraffin sections were prepared on P3 (6 and 24 h after insult) and on P12 (10 days after insult), respectively.

The brains were postfixed, dehydrated using graded alcohols, embedded in paraffin, and coronally sectioned (10-μm thick sections) from the genu of the corpus callosum to the end of the dorsal hippocampus. For immunohistochemical staining, four coronal sections, two at the striatum level (0.26 and 0.92 mm posterior to the bregma) and two at the dorsal hippocampus level (3.14 and 4.16 mm posterior to the bregma), per rat were selected [18] according to the reference planes in a rat brain atlas [22] and assessed for each brain.

For the cell counting (ED1+ microglia and MPO(+) neutrophils), myelin basic protein (MBP) score and integrated optical density (IOD) measurement (extravascular immunoglobulin G (IgG), glial fibrillary acidic protein (GFAP), and CXC chemokine ligand 5 (CXCL5)) after immunohistochemistry, three visual fields within the medial, middle, and lateral areas in the white matter of hemisphere per section of the four selected sections per brain as described were analyzed (Additional file 1).

Gray matter injury. Nissl-stained sections were scanned and the cross-sectional areas of the striatum, cortex, and hippocampus in the four brain sections described above were assessed manually by tracing the histological area using a computerized image analysis system (Image-Pro 6.0) linked to a Nikon E400 microscope. The total cross-sectional area in each brain region (cortex, striatum, and hippocampus) was then calculated in the sections assessed, and the percentage of area loss in the ipsilateral hemisphere versus the contralateral hemisphere was determined for each rat pup [18, 21].

White matter injury. MBP staining for assessing myelination and GFAP staining for assessing astrogliosis in the white matter were used as markers of white matter injury. After the permeabilization and blocking of nonspecific binding, sections were first incubated overnight at 4 °C with the anti-rat MBP monoclonal antibody (1:100; Millipore) or rabbit polyclonal anti-GFAP antibody (1:800; Millipore), rinsed, and incubated for 1 h at room temperature with the goat anti-rat (1:200; Santa Cruz) or anti-rabbit (1:300; Pierce Biotechnology) biotinylated secondary antibody. Positively stained cells were visualized using the avidin-biotin-peroxidase complex amplification (Pierce Biotechnology) and detected using diaminobenzidine tetrahydrochloride. MBP scores and GFAP signals were analyzed using ImagePro Plus 6.0. Measurements were performed at ×100 and ×200 magnification per visual field (0.579 and 0.145 mm^2^, respectively) for MBP scores and GFAP signals, respectively. Three visual fields in the medial, middle, and lateral areas of the white matter in each hemisphere per section and four sections per brain were analyzed and averaged [18, 23].

MBP expression was graded in the three areas within the white matter in each hemisphere per section using a 4-point scoring system, as described previously [18, 23]: 0, loss of processes and complete loss of capsule; 1, loss of processes with thinning or breaks in capsule; 2, complete loss of processes with intact capsule; 3, partial loss of processes; and 4, no MBP loss. The scores of each region were summed to obtain a total score (range, 0–12) for each hemisphere. Each section had a total MBP score in the ipsilateral and contralateral hemispheres. The mean IOD values for the GFAP signals in the white matter of the ipsilateral and contralateral hemispheres of each experimental group were compared with those of the control group to obtain the relative IOD ratios.

Ventricular size ratio. The measurement of the ipsilateral ventricle size ratio of the experimental group was modified from our previous study [24]. The ipsilateral ventricle size of area in the four brain sections (0.26, 0.92, 3.14, and 4.16 mm posterior to the bregma [22]) in each rat pup were assessed manually by tracing the ipsilateral ventricular area using a computerized image analysis system (Image-Pro 6.0) linked to the E400 microscope. The ipsilateral ventricle size ratio for each experimental rat was respectively calculated from the four brain sections: ipsilateral ventricle area in the experimental group/the respective ipsilateral ventricle area in the control group. The ipsilateral ventricle size ratios from the four sections were summed up and divided by 4 to obtain the ventricle size ratio for one rat of the experimental group. The averaged ipsilateral ventricle size ratio of the study group were the summed up of the ventricle size ratios from each rat in the same group and divided by the number of rats used in that group (Additional file 1).

### Assessment of neuroinflammation and BBB damage

At 6 and 24 h after HI, all the animals were perfused before the brains were removed and processed for immunohistochemical staining. The brains were postfixed, dehydrated using 30 % (*w*/*v*) sucrose in phosphate-buffered saline (PBS) for 2 days, and coronally sectioned (20 μm thick) from the genu of the corpus callosum to the end of the dorsal hippocampus. Four coronal sections, as described previously, were assessed for each brain. Immunoglobulin G (IgG) extravasation was used as an indicator of BBB permeability [18, 23, 25, 26], and MPO immunoreactive neutrophils were used for detecting neutrophil infiltration. Specific primary antibodies used were the rabbit anti-rat CXCL5 antibody (1:100; R&D Systems), mouse anti-rat ED1 antibody (1:100, Millipore), rabbit anti-rat MPO antibody (1:100; Abcam), and horseradish peroxidase-conjugated goat anti-rat IgG (1:200; Millipore). Biotinylated secondary antibodies were anti-rabbit and anti-mouse IgGs (all 1:200; Pierce Biotechnology). Biotin–peroxidase signals were detected using 0.5 mg/mL 3′3′-diaminobenzidine/0.003 % H_2_O_2_ (Dako, Carpinteria) as a substrate. Results were recorded using a microscope (BX51; Olympus).

Quantification was performed at ×400 magnification per visual field (0.0356 mm^2^) for CXCL5 signals and ED1(+) microglia and at ×200 magnification per visual field (0.145 mm^2^) for the MPO(+) neutrophils and extravascular IgG signals. Three visual fields within the medial, middle, and lateral areas in the white matter of each hemisphere per section and four sections per brain were analyzed and averaged. The mean IOD values in the white matter of the ipsilateral and contralateral hemispheres of each experimental group were compared with those of the control group to obtain the relative IOD ratios.

### Fluorescence immunocytochemical analyses

After blocking (×1 PBS, 2 % normal goat serum, and 0.1 % Triton X-100) for 1 h, the sections were incubated overnight at 4 °C with a mixture of any two of the following primary antibodies: anti-CXCL5 (1:50; abcam), anti-ED1 (1:100; Chemicon, Temecula, CA), anti-O4 IgM (1:100; Chemicon), anti-GFAP (1:200; Chemicon), anti-neuronal nuclear antigen (NeuN) (1:200; Millipore Bioscience Research Reagents), and anti-RECA (1:100; abcam). The sections were washed three times with 0.1 M PBS and incubated with Alexa Fluor 594 anti-mouse IgG/IgM or Alexa Fluor 488 anti-rabbit IgG (1:400; Invitrogen) for 1 h at room temperature. The fluorescence signals were detected, and the results were recorded using a microscope (E400; Nikon Instech) at excitation–emission wavelengths of 596–615 nm (Alexa Fluor 594, red) and 470–505 nm (Alexa Fluor 488, green) [18, 23].

### Western blot analysis

The rat pups were sacrificed 3, 6, and 24 h after insult for Western blot analysis. Ipsilateral cerebral white matter tissues were homogenized in a cold lysis buffer, and the protein concentrations were determined using a Bio-Rad Protein Assay kit (Bio-Rad Laboratories). Samples were separated using 18 % SDS-PAGE and blotted electrophoretically onto polyvinylidene fluoride membranes. The membranes were incubated with primary antibodies: anti-rat CXCL5 (1:400; R&D Systems) or anti-actin (1:1000; Chemicon). Immunoreactivity was detected using the horseradish peroxidase-conjugated anti-mouse IgG antibody (1:10,000; Calbiochem) and visualized using enhanced chemiluminescence (Pierce Biotechnology). VisionWorks LS analysis software (Ultra-Violet Products Ltd) was used for densitometry analysis, and CXCL5 levels were assessed after they were normalized to actin expression [21].

### Statistical analysis

Statistical significance (*p* < 0.05) was determined using the Kruskal–Wallis test, and Dunn’s method was used for post hoc comparisons. Continuous data were presented as means ± standard errors of the mean (SEMs).

## Results

### LPS-sensitized HI caused microglial activation, BBB damage, and white matter injury

**Fig. 1:**
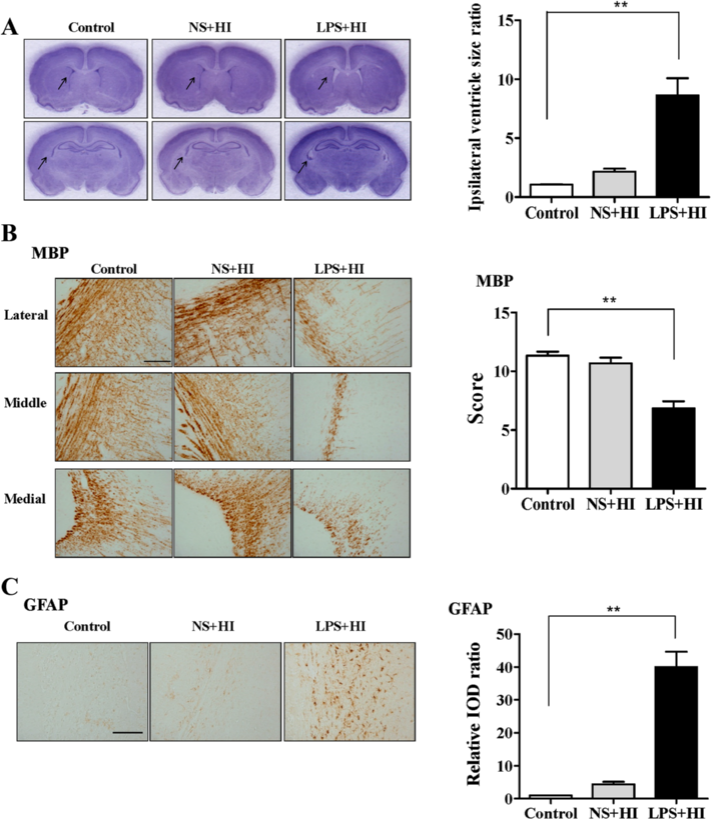
LPS-sensitized HI caused selective white matter injury. P2 rat pups received LPS (0.05 mg/kg) or NS 3 h before 90 min of HI. Neuropathological tests were performed on P12 (**a**, *left panel*). Nissl staining revealed no significant injury in the gray matter of the control (*n* = 6), NS + HI (*n* = 6), and LPS + HI (*n* = 6) groups. Upper panel: brain section at the level of striatum (0.26 mm posterior to the bregma) and lower panel: the section at the level of dorsal hippocampus (3.14 mm posterior to the bregma). The LPS + HI group had significantly higher ipsilateral ventricular size ratios (**a**, right panel), lower myelination (MBP) (**b**), and higher astrogliosis (GFAP) (**c**) in the white matter than those of the control group. *Scale bar* = 100 μm for MBP and GFAP. Values are means ± SEMs. **p < 0.01. Values are means ± SEMs. NS, normal saline; LPS, lipopolysaccharide; HI, hypoxic ischemia; MBP, myelin basic protein; GFAP, glial fibrillary acidic protein

**Fig. 2:**
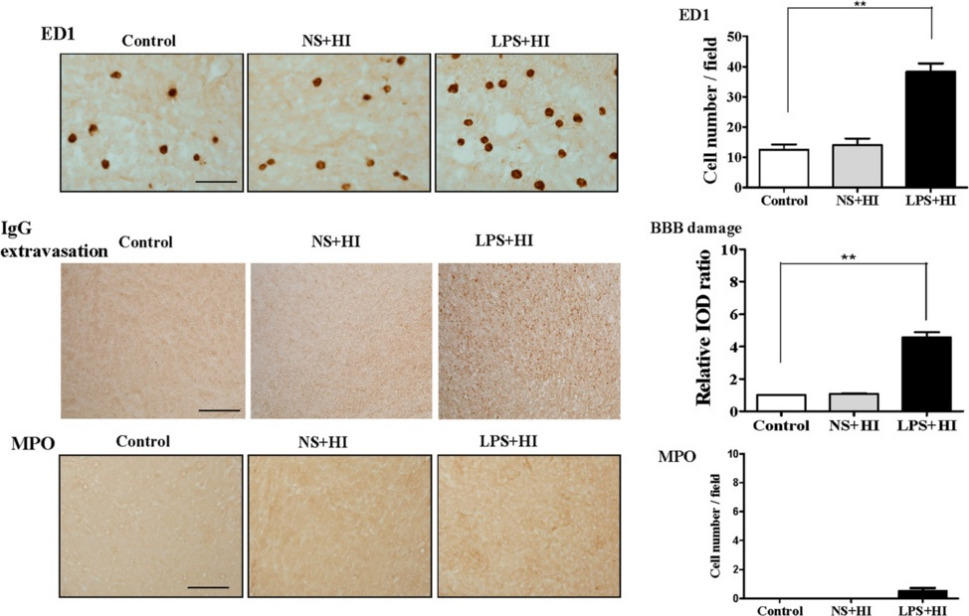
LPS-sensitized HI induced microglial activation and BBB damage. Neuroinflammation and BBB damage were assessed 24 h after insult. The LPS + HI group had a significantly higher number of ED1(+) activated microglia (*upper panel*) and significantly greater IgG extravasation suggesting BBB damage (*middle panel*) but fewer MPO(+) neutrophils (*lower panel*) in the white matter than did the control group (*n* = 6 in each group). BBB, blood–brain barrier. *Scale bar* = 50 μm (ED1) and = 100 μm (IgG and MPO). Values are means ± SEMs, **p < 0.01

We first established the sensitization effect of LPS on HI-induced white matter injury in the P2 rat pups. On P12, Nissl staining revealed no significant neuronal damage in the cortex, striatum, and hippocampus in the NS + HI and LPS + HI groups (Fig. 1a, left panel). By contrast, the LPS + HI group, but not the NS + HI group, had significantly higher ipsilateral ventricular size ratios (Fig. 1a, right panel), lower myelination (MBP staining, Fig. 1b), and higher astrogliosis (GFAP staining, Fig. 1c) in the white matter of the ipsilateral cerebral hemisphere than those of the control group. Resting microglia were identified as ramified microglia with long processes, whereas primed or activated microglia were identified as microglia that were more rounded, with retracted and shorter processes. At 24 h after insult, the LPS + HI, but not the NS + HI group, had a significantly higher number of ED1(+) activated microglia (Fig. 2, upper panel) and significantly increased BBB damage (Fig. 2, middle panel) but fewer MPO(+) neutrophils (Fig. 2, lower panel) in the white matter compared with the control group.

### LPS-sensitized HI upregulated CXCL5 expression in the microglia and vascular endothelial cells in the white matter

**Fig. 3:**
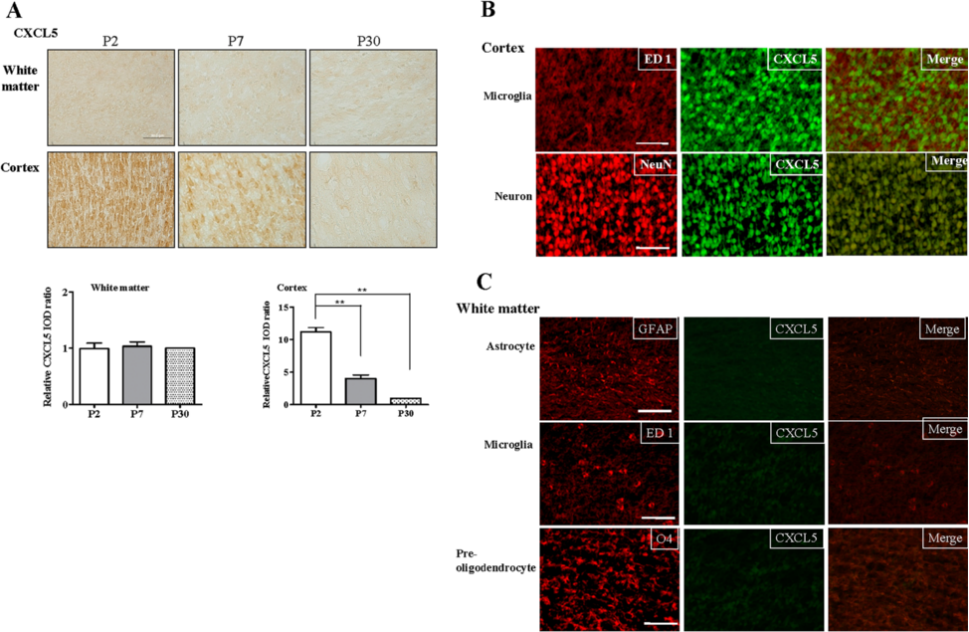
CXCL5 expression in the normal developing rat brain. **a** CXCL5 immunohistochemical analysis demonstrated that P7 (*n* = 4) and, particularly, P2 pups (*n* = 5) had significantly higher CXCL5 expression in the cerebral cortex than that of P30 rats (*n* = 4). Values are means ± SEMs. The P2 pups had significantly higher CXCL5 expression in the cerebral cortex than that of the P7 pups and P30 rats. CXCL5 expression was low in the white matter, and no significant difference was observed between the three groups. **b** Immunofluorescence staining in the P2 pups revealed that the most CXCL5-positive cells in the cortex were neurons (NeuN) rather than microglia (ED1), and CXCL5 expression was absent in all white matter cells. **c** *Scale bar* = 50 μm

In the normal developing brain of the P7 and, particularly, P2 pups, significantly higher CXCL5 expression was observed in the cerebral cortex compared with that of the P30 rats (Fig. 3a). By contrast, the CXCL5 expression was low in the white matter, and no significant difference was observed between the P2, P7, and P30 rats. Immunofluorescence of the P2 cortex revealed that most of the CXCL5^+^ cells were NeuN(+) neurons and not microglia (Fig. 3b). By contrast, CXCL5 was not expressed in any white matter cells (Fig. 3c).

**Fig. 4:**
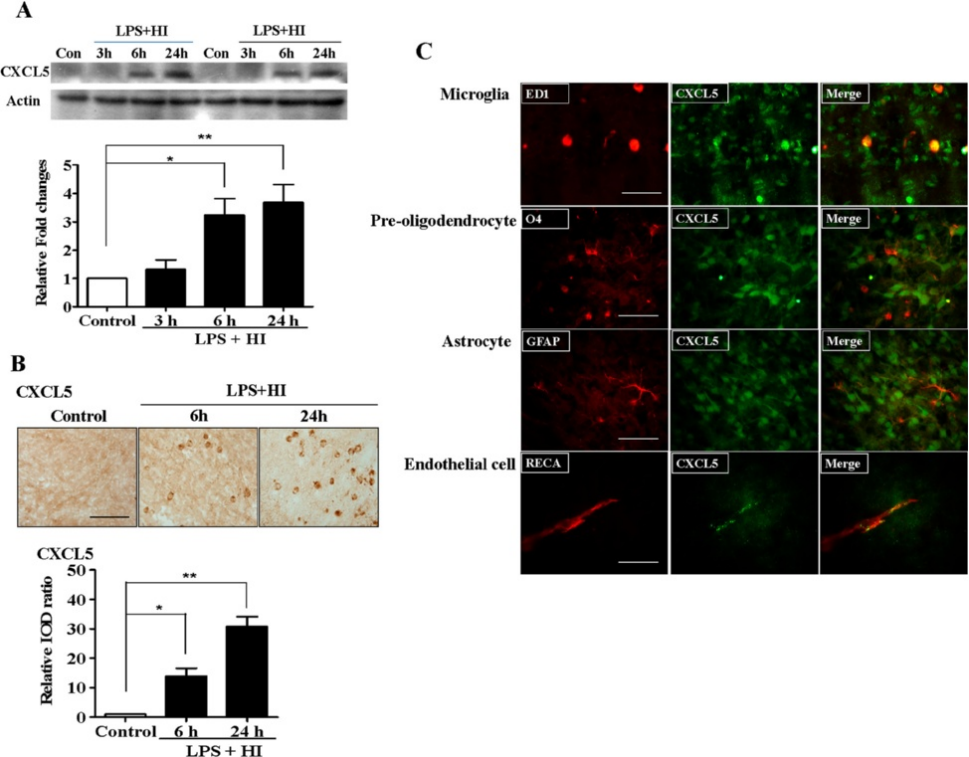
CXCL5 upregulation in the white matter after LPS-sensitized HI. **a** Western blots of the white matter revealed significant increases in CXCL5 levels at 6 and 24 h after LPS + HI (*n* = 4 in each study group). **b** Immunohistochemical analyses revealed that the LPS + HI group had significantly increased CXCL5 immunoreactivity in the white matter at 6 h and, particularly, 24 h after HI compared with the control group (*n* = 6 in each study group). *Scale bar* = 50 μm. Values are means ± SEMs. **c** Immunofluorescence analysis of the white matter 24 h after LPS + HI revealed that CXCL5 was upregulated mainly in activated microglia (ED1) and vascular endothelial cells (RECA) and not in astrocytes (GFAP) or preoligodendrocytes (O4). *Scale bar* = 50 μm. *p < 0.05, **p < 0.01

Western blots of the white matter revealed that the LPS + HI group had significantly higher CXCL5 expression at 6 and 24 h after HI than that of the controls (Fig. 4a). Immunohistochemical analyses confirmed that the LPS + HI group had significantly higher CXCL5 immunoreactivity in the white matter at 6 h and, particularly, 24 h after HI than that of the control group (Fig. 4b). Immunofluorescence of the white matter 24 h after LPS-sensitized HI revealed that CXCL5 was upregulated mainly in the ED1(+) microglia and RECA(+) vascular endothelial cells and not in astrocytes or O4(+) preoligodendrocytes (Fig. 4C).

### CXCR2 inhibition attenuated microglial activation, reduced BBB damage, and protected against white matter injury after LPS-sensitized HI

**Fig. 5:**
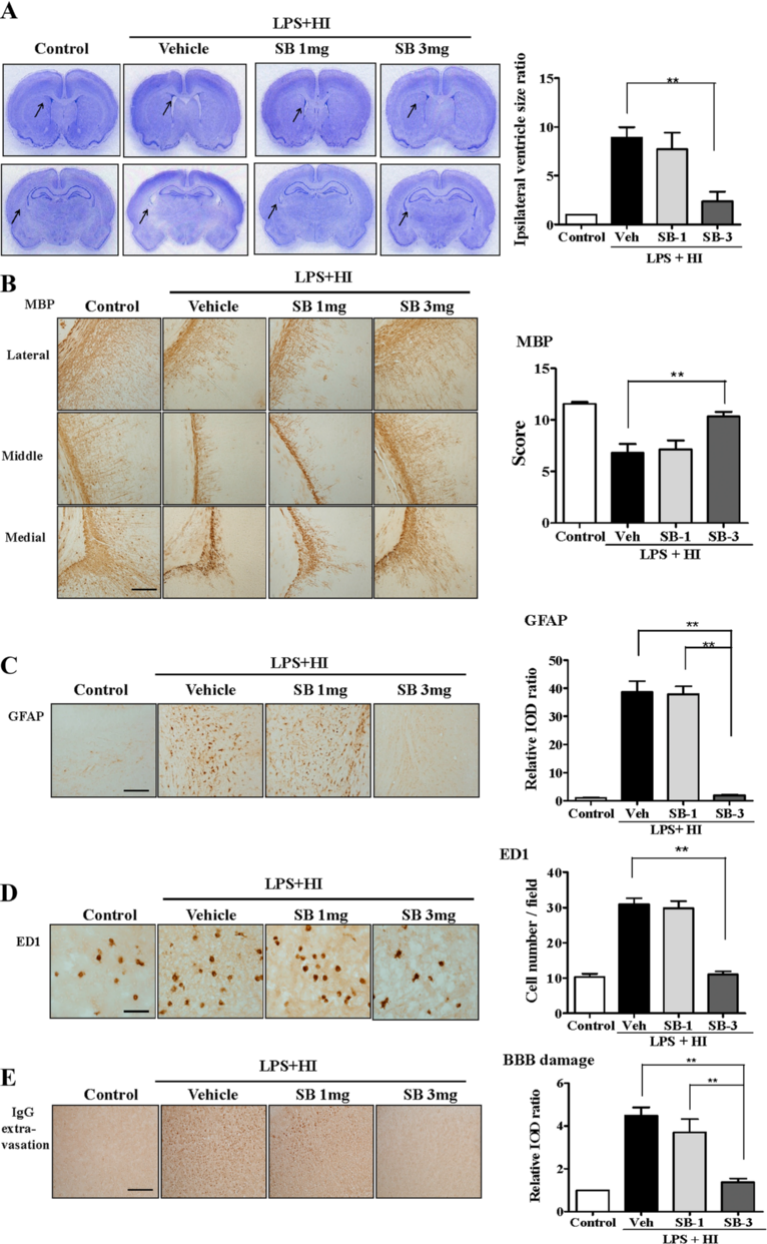
Pharmacological inhibition of CXCR2 significantly attenuated microglial activation and BBB damage and protected against white matter injury after LPS-sensitized HI. A selective nonpeptide inhibitor of CXCR2, SB22 5002, was used to examine the role of the CXCL5–CXCR2 pathway in white matter injury after LPS-sensitized HI. After LPS + HI on P2, the SB-3 group (*n* = 9), but not the SB-1 group (*n* = 7), had significantly reduced ipsilateral ventricular size ratios (**a**), increased myelination (MBP) (**b**), and reduced astrogliosis (GFAP) (**c**) in the white matter on P12 compared with the vehicle-treated pups (*n* = 9). *Scale bar* = 100 μm. The SB-3 group (*n* = 9), but not the SB-1 group (*n* = 7), had a significantly reduced number of activated microglia (ED1) (**d**) and significantly lower BBB damage (IgG extravasation) (**e**) in the white matter compared with the vehicle group (*n* = 9) at 24 h after HI. *Scale bar* = 50 μm (ED1) and 100 μm (IgG); values are means ± SEMs, **p < 0.01

We used SB225002, a selective nonpeptide inhibitor of CXCR2, for examining the role of the CXCL5–CXCR2 pathway in white matter injury after LPS-sensitized HI. On P12, the SB-3 group, but not the SB-1 group, had significantly lower ipsilateral ventricular size ratios (Fig. 5a), higher MBP scores (Fig. 5b), and lower astrogliosis (Fig. 5c) in the white matter than those of the vehicle-treated group. The SB-3 group, but not the SB-1 group, had a significantly lower number of ED1(+) activated microglia (Fig. 5d) and significantly reduced BBB damage (Fig. 5e) in the white matter 24 h after insult compared with the vehicle group. These results suggest that the CXCL5–CXCR2 pathway plays an important role in microglial activation, BBB damage, and subsequent white matter injury after LPS-sensitized HI in the immature brain.

### CXCL5-sensitized HI caused microglial and neutrophil infiltration, BBB damage, and white matter injury

**Fig. 6:**
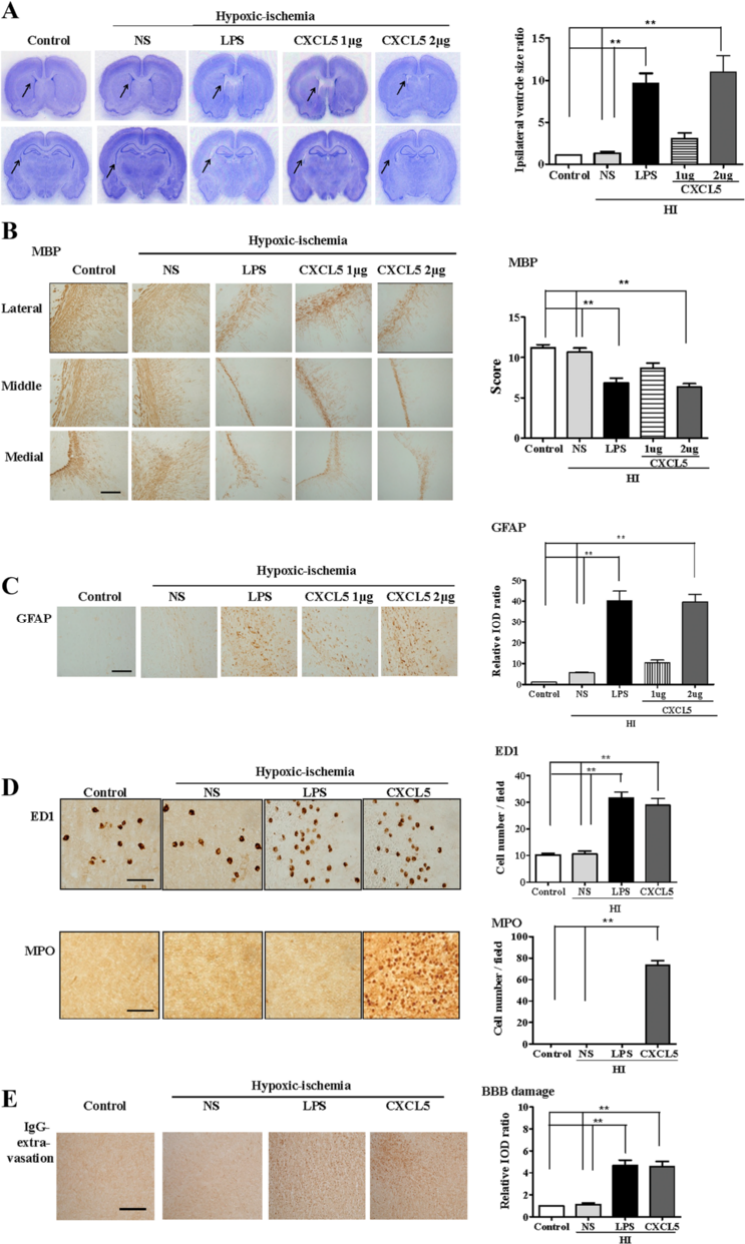
CXCL5-sensitized HI induced microglial activation, neutrophil infiltration, and BBB damage and caused white matter injury. Intracerebroventricular infusion of recombinant CXCL5 (1 or 2 μg) or NS followed by HI on P2 showed that the 2-μg CXCL5 + HI group (*n* = 6), but not the 1-μg CXCL5 + HI group (*n* = 6), had significantly higher ipsilateral ventricle size ratios (**a**), lower myelination (MBP) (**b**), and higher astrogliosis (GFAP) (**c**) in the white matter compared with the NS (*n* = 6) and the control groups (*n* = 6) on P12. *Scale bar* = 100 μm. At 24 h after HI, the 2-μg CXCL5 + HI (*n* = 6) and LPS + HI groups (*n* = 6) had a significantly higher number of activated microglia (ED1) (**d**) and BBB damage (**e**) in the white matter compared with the NS (*n* = 6) and control groups (*n* = 6). The 2-μg CXCL5 + HI group, but not the LPS + HI group, had significantly increased neutrophil infiltrations (MPO) (**d**) in the white matter. *Scale bar* = 50 μm (ED1) and 100 μm (IgG and MPO); values are means ± SEMs, **p < 0.01

Subsequently, we examined whether CXCL5-sensitized HI caused white matter injury in P2 pups. On P12, the 2-μg CXCL5 + HI group, but not the 1-μg CXCL5 + HI group, had significantly higher ipsilateral ventricle size ratios (Fig. 6a), lower MBP expression (Fig. 6b), and higher astrogliosis (Fig. 6c) in the white matter area compared with the NS + HI or control group. Twenty-four hours after HI, the 2-μg CXCL5 + HI and LPS + HI groups had a significantly higher number of ED1(+) microglia (Fig. 6d) and significantly greater BBB damage (Fig. 6e) in the white matter compared with the NS + HI and control groups. By contrast, the 2-μg CXCL5 + HI group, but not the LPS + HI group, had significantly higher MPO(+) neutrophil infiltration (Fig. 6d) in the white matter than that of the NS + HI and control groups. These results suggested that along with HI, CXCL5 induced microglial activation and neutrophil infiltration, increased BBB damage, and caused white matter injury.

### CXCL5 alone increased neutrophil infiltration and BBB damage and caused white matter injury

**Fig. 7:**
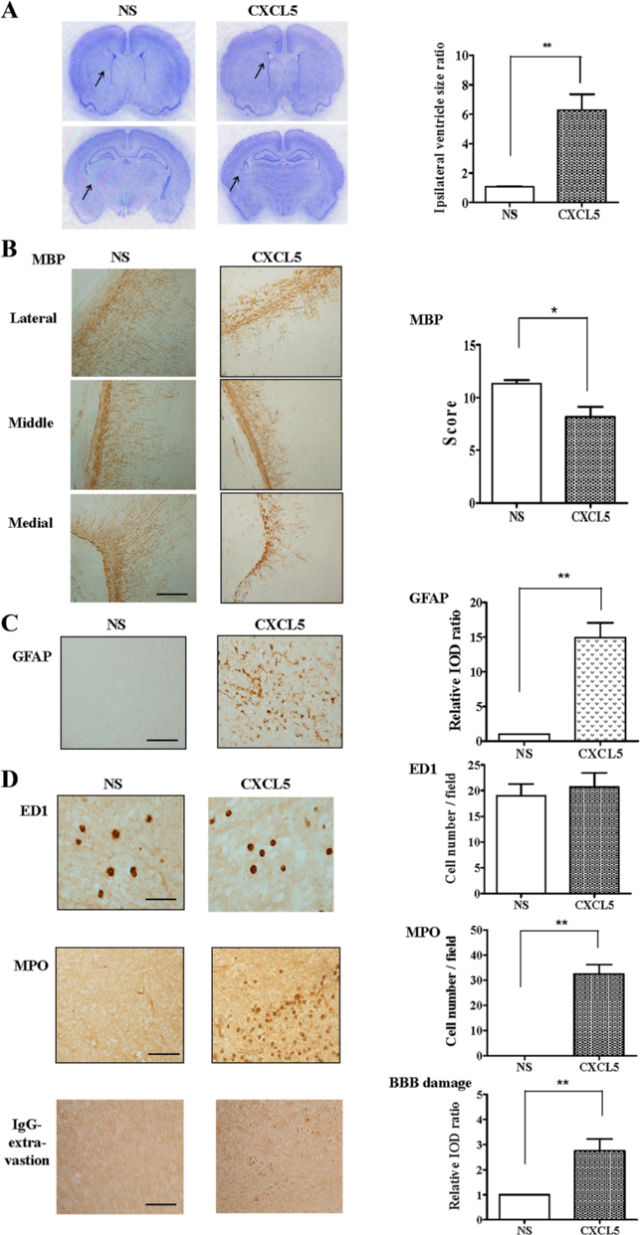
CXCL5 alone increased neutrophil infiltration and BBB damage and caused white matter injury. Intracerebroventricular infusion of recombinant CXCL5 (2 μg) or NS on P2 revealed that the CXCL5 group (*n* = 6) had significantly higher ipsilateral ventricle size ratios (**a**), reduced myelination (MBP) (**b**), and increased astrogliosis (GFAP) (**c**) in the white matter on P12 compared with the vehicle group (*n* = 6). *Scale bar* = 100 μm. (**d**) At 24 h after injection, the NS (*n* = 6) and CXCL5 groups (*n* = 6) showed no detectable ED1(+) activated microglia in the white matter. By contrast, the CXCL5 group had a significantly higher number of MPO(+) neutrophils and significantly greater BBB damage compared with the vehicle group. *Scale bar* = 50 μm (ED1) and 100 μm (IgG and MPO); values are means ± SEMs, *p < 0.05, **p < 0.01

We examined whether CXCL5 alone induced white matter injury in the P2 pups. On P12, the CXCL5 group had significantly higher ipsilateral ventricle size ratios (Fig. 7a), lower MBP expression (Fig. 7b), and higher astrogliosis (Fig. 7c) in the white matter than those of the vehicle group. At 24 h postinjection, no detectable ED1(+) microglia (Fig. 7d, upper panel) were observed in the white matter of the NS and CXCL5 groups. By contrast, the CXCL5 group had a significantly higher number of MPO(+) neutrophils (Fig. 7d, middle panel) and greater BBB damage (Fig. 7d, lower panel) compared with the vehicle group. These findings suggested that CXCL5 alone was sufficient to induce significant neutrophil accumulation and BBB damage and cause white matter injury.

## Discussion

HI and inflammation are the two major pathogenetic mechanisms of white matter injury in very preterm infants [7, 17, 27]. Our previous study [18] on P2 rat pups had demonstrated that low-dose LPS or 90-min HI alone caused no significant white matter injury. The LPS-treated or 90-min HI pups also showed no evidence of microglial activation or BBB breakdown in the white matter. In the present study, LPS-sensitized HI induced microglial activation and BBB damage in the P2 rat pups and caused long-term white matter injury (increased ipsilateral ventricular size and astrogliosis and decreased myelination). After LPS-sensitized HI, the CXCL5 levels were markedly upregulated in the white matter and expressed mainly in the microglia and vascular endothelial cells. Attenuating CXCL5 function by inhibiting CXCR2 significantly attenuated microglial activation, reduced BBB damage, and protected against white matter injury. Furthermore, CXCL5 administration with HI or CXCL5 alone caused significant BBB damage and white matter injury in association with different neuroinflammatory mechanisms. CXCL5 with HI induced microglial activation and neutrophil infiltration, whereas CXCL5 alone caused predominant neutrophil infiltration. These results suggest that CXCL5-mediated signaling pathway plays an essential role in neuroinflammatory activation, BBB disruption, and subsequent white matter injury in the immature brain.

Neuroinflammation and BBB disruption have been associated with the severity of HI injury in the immature brain [7–9, 18, 23, 28, 29]. Neuroinflammation is the neuropathological hallmark of white matter injury in preterm infants [7, 29] and may exacerbate injury through BBB damage [9, 30]. Inflammation involves microglial and endothelial cell activation, leading to proinflammatory cytokine and endothelial adhesion molecule secretion, and is associated with vascular tight junction modification [30]. The damaged microvessels may recruit activated leukocytes at the injured site of the brain through the disrupted BBB, leading to sustained neuroinflammation, which in turn further damages the brain [9, 29, 30]. Neuroinflammation and BBB damage may be two mutually potentiating mechanisms for white matter injury in the developing brain. Our study demonstrated for the first time that LPS-sensitized HI-induced CXCL5 expression, CXCL5 in association with HI, and CXCL5 alone play essential roles in neuroinflammation, BBB damage, and subsequent white matter injury in the immature rat brain.

Chemokines rapidly secreted within the central nervous system in response to injuries and infections contribute to host defense mechanisms and disease progression. CXCL5 released from endothelial cells [31] and inflammatory monocytes [4] can propagate the proinflammatory process through the recruitment and activation of additional cellular mediators of inflammation, such as macrophages and neutrophils. Aberrant CXCL5 levels have been detected in various acute and chronic diseases, such as microbial infections, rheumatoid arthritis, obesity, and inflammatory bowel diseases [3, 4, 32]. Elevated CXCL5 expression was observed in many complications of preterm infants [11, 13, 14]. High CXCL5 levels were observed in clinical chorioamnionitis of preterm births [11] and in surgically resected tissue samples of necrotizing enterocolitis from preterm infants [14]. In addition, preterm infants with moderate or severe bronchopulmonary dysplasia had significantly higher CXCL5 levels in the gastric fluid than those with no or mild bronchopulmonary dysplasia did [13]. Currently, information regarding the role of CXCL5 in white matter injury among preterm infants is scant. Our experimental study provides the first evidence that CXCL5 is markedly upregulated and causes white matter injury after LPS-sensitized HI in P2 immature rat brains. In addition, CXCL5, in association with HI or alone, causes marked white matter injury. These findings suggest that CXCL5 may play a critical role in the pathogenesis of white matter injury of the immature brain. Additional clinical studies are warranted to test whether CXCL5 is an essential biomarker predicting the occurrence of white matter injury and its long-term outcome in preterm infants.

Blocking CXCL5 activity using the CXCR2 antagonist SB225002 is highly effective in ameliorating acute experimental colitis [20] and ischemic brain injury [33]. CXCR2 is a chemokine receptor expressed on endothelial cells, oligodendrocytes, and various immune cells [34] and is essential for neutrophil recruitment at tissue injury- or infection-induced inflammation sites. CXCR2 and its ligands have been implicated in several inflammation-mediated diseases. CXCR2 has been associated with inflammation and myelin disorders [2]. Antagonism of the chemokine receptor CXCR2 has been proposed as a therapeutic strategy for inflammatory diseases [34]. Furthermore, the role of CXCR2 in myeloid and nonmyeloid cells in association with the destruction and repair of myelin has been demonstrated in an animal model of demyelination [35]. Myelin repair was accelerated by inactivating CXCR2 on nonhematopoietic cells [35]. In our experimental study, CXCL5 levels were markedly upregulated, particularly in the microglia and vascular endothelial cells of the white matter of the immature brain after LPS-sensitized HI. CXCL5 upregulation in the microglia and vascular endothelial cells may contribute to neuroinflammation and BBB damage and subsequent white matter injury, potentially through CXCR2, because attenuating CXCL5 activity by inhibiting CXCR2 significantly attenuated neuroinflammation and BBB damage and protected against white matter injury in the immature brain. Furthermore, after LPS-sensitized HI, CXCL5 is a potent chemoattractant for microglia, which may enhance neuroinflammation by binding and signaling through its receptor CXCR2 and further disrupting the BBB integrity. These findings suggest that CXCL5–CXR2 signaling is not only the shared mechanism of neuroinflammation and BBB disruption but also a promising drug target for clinical white matter injury in the immature brain.

In the mitogen-activated protein kinase family, c-Jun N-terminal kinases (JNKs) are crucial stress-responsive kinases that control essential processes, such as inflammation and apoptosis. JNK signaling is triggered by extracellular signals, such as cytokines and environmental stresses [36]. Our previous studies showed that tumor necrosis factor-alpha (TNF-α)-mediated TNF receptor 1 signaling was an upstream pathway leading to JNK activation that mediated neuroinflammation and BBB damage and induced white matter injury in the immature brain [18, 23]. CXCL5 can regulate the production of TNF-α and the activation of the NF-kB and MAPK pathways, including the JNK pathway [37]. Our study findings suggest that CXCL5 signaling may be an upstream pathway leading to JNK activation in the immature brain.

In addition to regulating inflammatory cell migration, chemokines are valuable in normal human brain development [38]. In this study, the CXCL5 expression in the neurons of the cerebral cortex of the P2 rat pups was higher than that of the P7 and P30 rats. The regional distribution of CXCL5 in the developing cerebral cortex may be related to the proliferation, migration, and differentiation of various cell types in the developing central nervous system [38]. All normal P2, P7, and P30 rats had extremely low CXCL5 expression in the white matter. By contrast, high CXCL5 expression was observed after selective white matter injury induced by LPS-sensitized HI, indicating that CXCL5 may be a critical tissue injury response marker.

We and others have used IgG extravasation in the perfused brain as an indicator of BBB damage in the neonatal brain [18, 23, 25, 26]. All the animals were perfused before the brain were removed and processed for IgG staining. Extravascular IgG immunoreactivity in the brain after HI is located at cellular as well as parenchymal levels. IgG entry into the brain cells after ischemia has been described in studies using immunostaining [39–41]. Glia can rapidly take up plasma proteins from the extracellular space of the injured brain through endocytosis [41]. Fc-receptors on reactive microglia may trap IgG in the tissue to facilitate its phagocytic activity [42]. In addition, extravasated plasma constituents after cerebral ischemia may act also as an inductive factor on microglial cells [43]. Additional sensitive markers for BBB damage are needed for more comprehensive assessment of BBB integrity in future studies of the neonatal brain injury.

## Conclusions

**Fig. 8:**
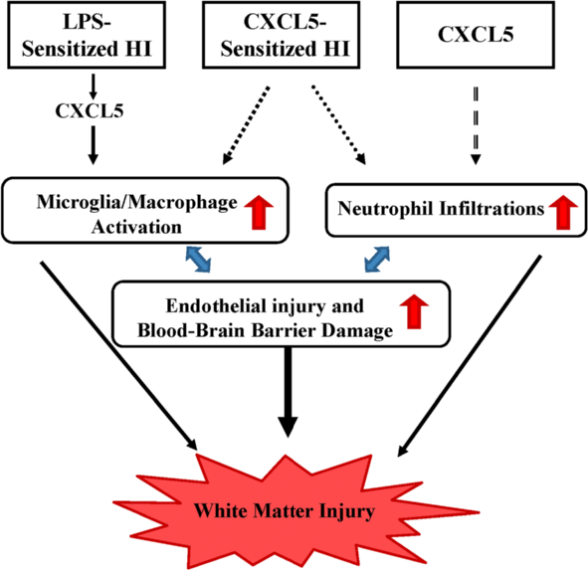
Diagram showing that the proposed CXCL5-mediated signaling pathway, triggered by LPS-sensitized HI, CXCL5-sensitized HI, or CXCL5 alone, plays a crucial role in neuroinflammation and BBB disruption and subsequent white matter injury in the immature brain. Neuroinflammation and BBB damage are two mutually potentiating mechanisms leading to sustained neuroinflammation and BBB disruption in white matter injury of the developing brain. White matter injury can be induced by different neuroinflammatory mechanisms: predominant microglial activation by LPS-sensitized HI, microglial activation and neutrophil infiltration by CXCL5-sensitized HI, and predominant neutrophil infiltration by CXCL5 alone

White matter injury in preterm infants may not be associated with a single etiological pathway but may instead represent a generic tissue injury response of the white matter at a specific stage of development to various insults. Excessive recruitment of leukocytes is critical to the pathogenesis of white matter injury, and the magnitude and duration of the inflammatory process may ultimately determine the outcome of preterm infants with white matter injury. According to the results of our study, CXCL5 plays a central role as a converging point for upstream HI or infection and downstream neuroinflammation and BBB damage in the pathogenesis of white matter damage in the immature brain (Fig. 8). Our study findings suggest that CXCL5 may be a potential biomarker for white matter injury and cerebral palsy in preterm infants. Moreover, pharmacologically blocking CXCL5 signaling or its cognate receptor CXCR2 to attenuate dysregulated neuroinflammation is a potential strategy for treating white matter injury in the immature brain.

## Additional file

Additional file 1:
**Supplementary methods.** The measurement of cerebral ventricular size and quantification methods for gray matter injury and immunohistochemistry are described in detail here. (DOC 5563 kb)

## Abbreviations

BBB: blood–brain barrier
GFAP: glial fibrillary acidic protein
HI: hypoxic ischemia
LPS: lipopolysaccharide
IgG: immunoglobulin G
IOD: integrated optical density
JNK: c-Jun N-terminal kinase
TNF-α: tumor necrosis factor-alpha
MBP: myelin basic protein
NS: normal saline
