# Supplementary material for: CXCL5 signaling is a shared pathway of neuroinflammation and blood–brain barrier injury contributing to white matter injury in the immature brain
Source: J Neuroinflammation. 2016 Jan 6;13:6. doi: 10.1186/s12974-015-0474-6 (PMC4704424; doi:10.1186/s12974-015-0474-6)
Supplement: Additional file 1: — Supplementary methods. The measurement of cerebral ventricular size and quantification methods for gray matter injury and immunohistochemistry are described in detail here. (DOC 5563 kb) [file 12974_2015_474_MOESM1_ESM.doc]

**Material and Method**

**Ventricular size ratio.**

The measurement of the ipsilateral ventricle size ratio of the experimental group was modified from our previous study [24]. The ipsilateral ventricle size of area in the four brain sections (0.26 mm, 0.92 mm, 3.14 mm and 4.16 mm posterior to the bregma [22]) in each rat pup were assessed manually by tracing the ipsilateral ventricular area using a computerized image analysis system (Image-Pro 6.0) linked to the E400 microscope. The ipsilateral ventricle size ratio for each experimental rat was respectively calculated from the four brain sections = ipsilateral ventricle area in the experimental group / the respective ipsilateral ventricle area in the control group. The ipsilateral ventricle size ratios from the four sections were summed up and divided by 4 to obtain the ventricle size ratio for one rat of the experimental group. The averaged ipsilateral ventricle size ratio of the study group were the summed up of the ventricle size ratios from each rat in the same group and divided by the number of rats used in that group.

Representative brain section of 0.26 mm posterior to the bregma is used to demonstrate calculation of ipsilateral ventricle size ratio of the experimental rat: B/A


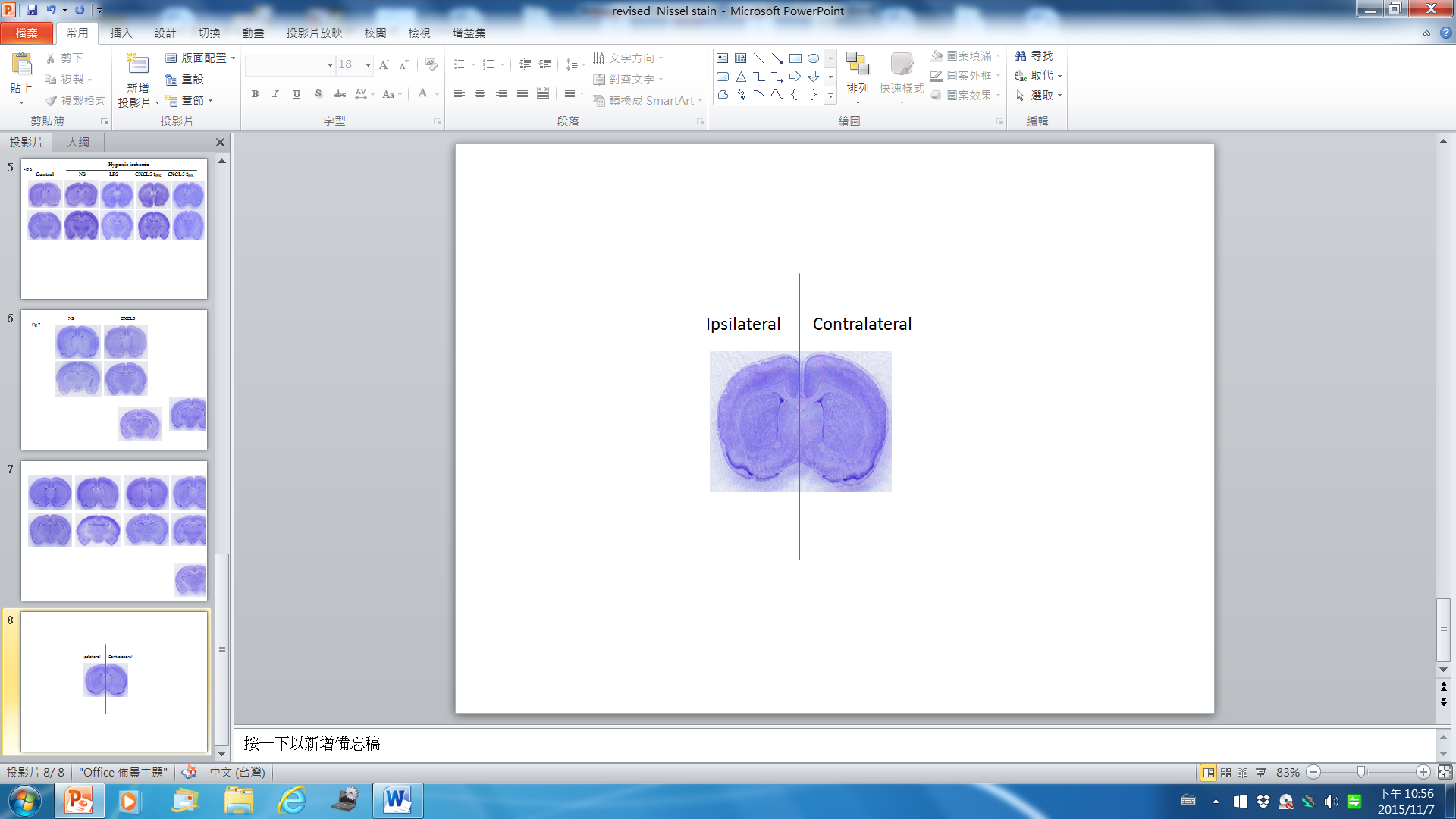


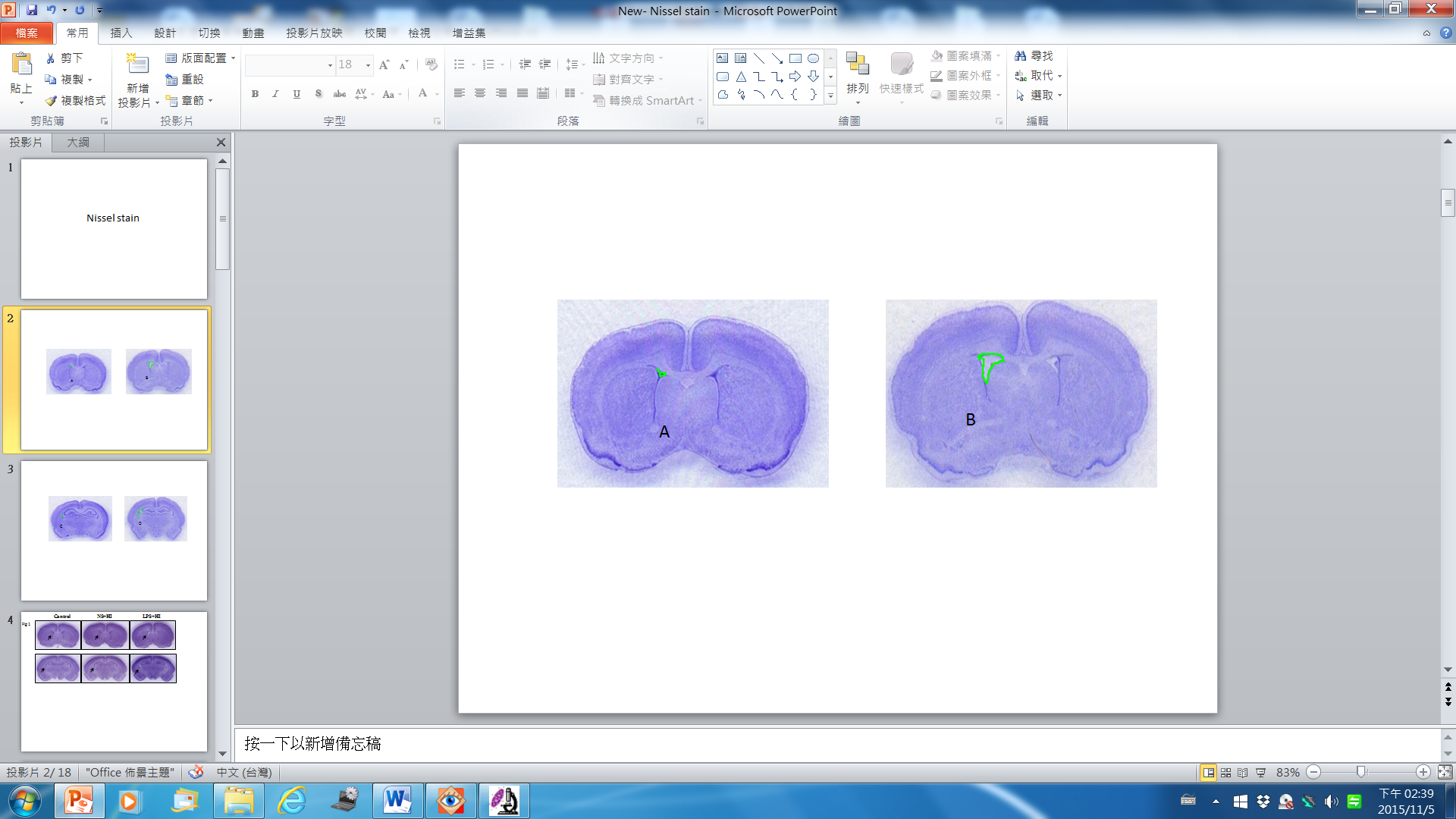


Representative brain section of 3.14 mm posterior to the bregma is used to demonstrate calculation of ipsilateral ventricle size ratio of the experimental rat: D/C


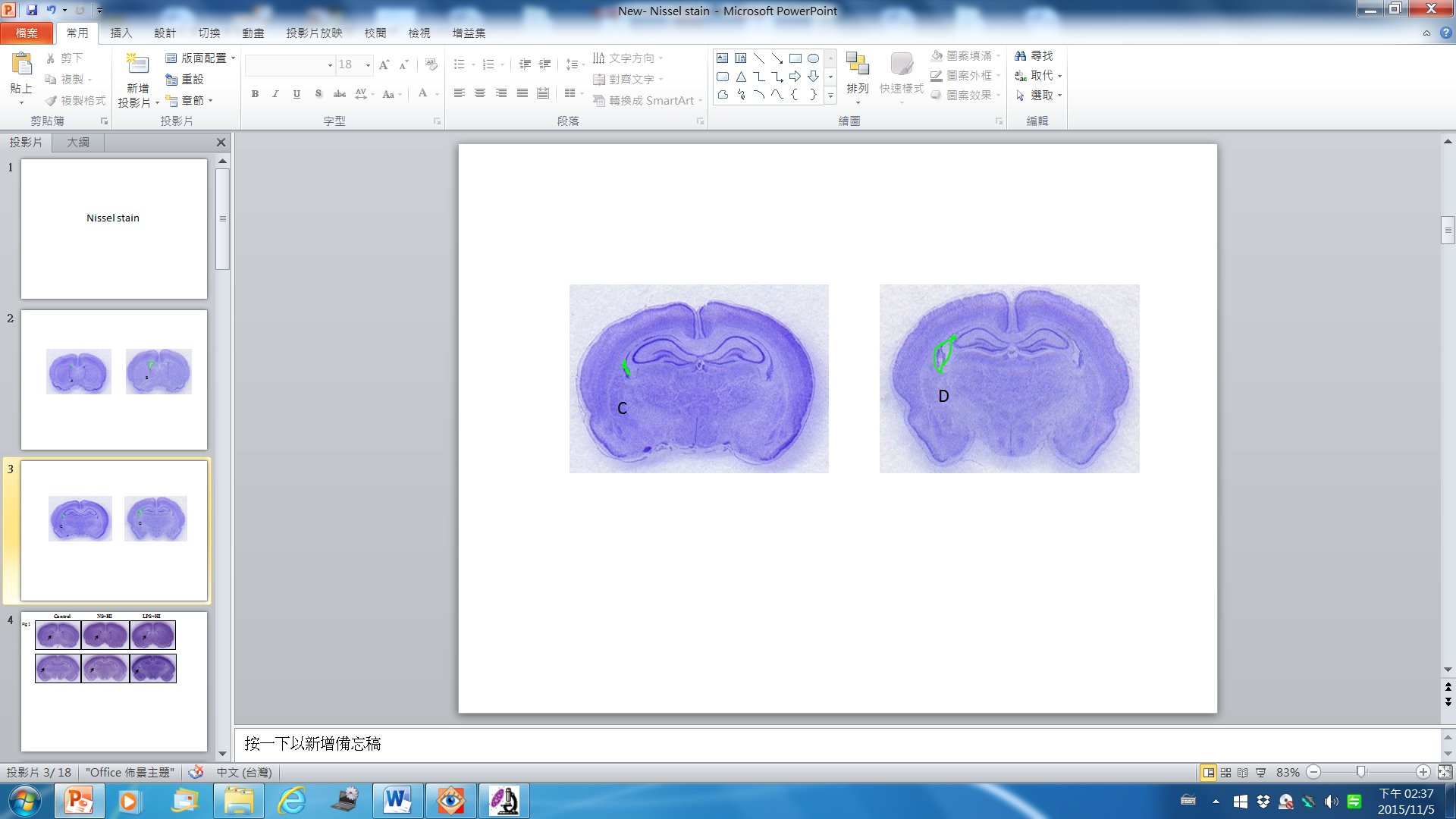


**Gray matter injury.**

Nissl-stained sections were scanned and the cross-sectional areas of the striatum, cortex, and hippocampus in the four brain sections described above were assessed manually by tracing the histological area using a computerized image analysis system (Image-Pro 6.0) linked to a Nikon E400 microscope. The total cross-sectional area in each brain region (cortex, striatum and hippocampus) was then calculated in the sections assessed, and the percentage of area loss in the ipsilateral hemisphere versus the contralateral hemisphere was determined for each rat pup [18,21].

Area loss in the cortex was assessed in the four coronal sections (0.26 mm, 0.92 mm, 3.14 mm and 4.16 mm posterior to the bregma) according to the reference planes in a rat brain atlas [22] of each rat pup. Area loss in the striatum was measured in two coronal sections (0.26 mm and 0.92 mm posterior to the bregma) and area loss in the hippocampus was examined in two coronal sections (3.14 mm and 4.16 mm posterior to the bregma).

Representative brain section at 0.26 mm posterior to the bregma was used to assess the cortex area loss: Percentage of cortex area loss = Ipsilateral cortex area (A) / Contralateral cortex area (B)


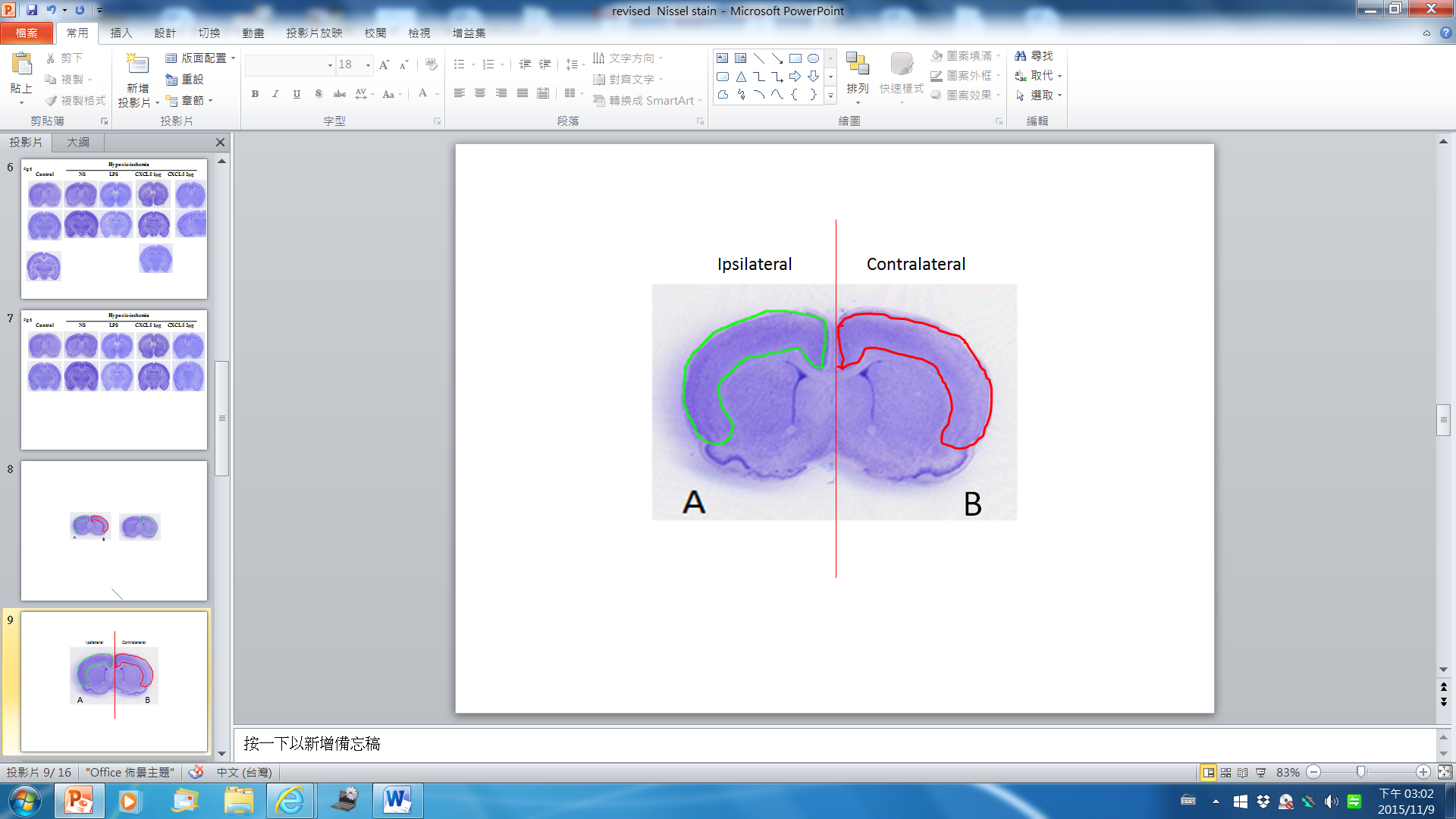


Representative brain section at 0.26 mm posterior to the bregma was used to assess the striatum area loss: Percentage of striatum loss = Ipsilateral striatum area (A) / Contralateral striatum area (B)


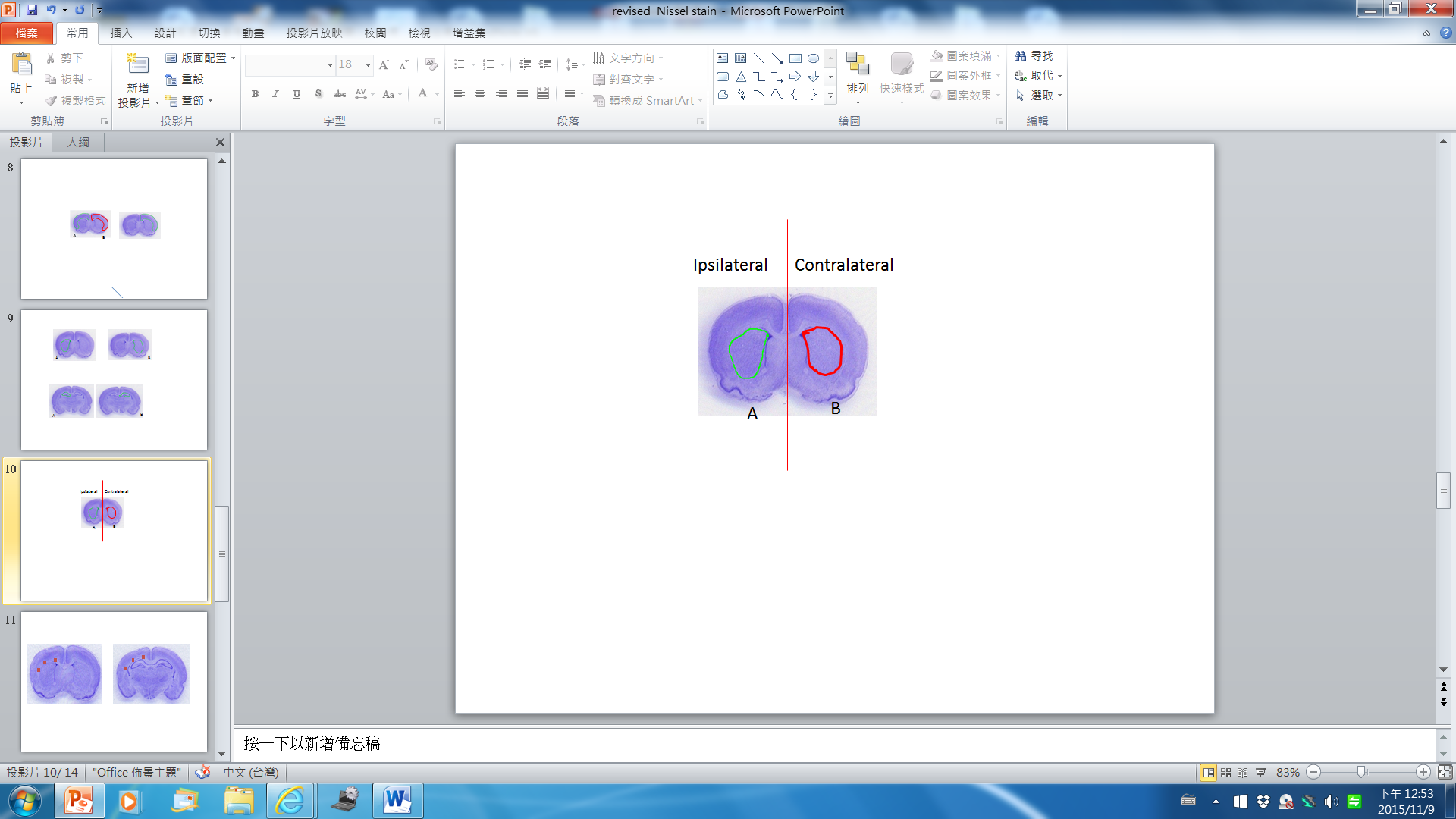


Representative brain section at 3.14 mm posterior to the bregma was used to assess hippocampus area loss: Percentage of hippocampus area loss = Ipsilateral hippocampus area (A) /contralateral hippocampus area (B)


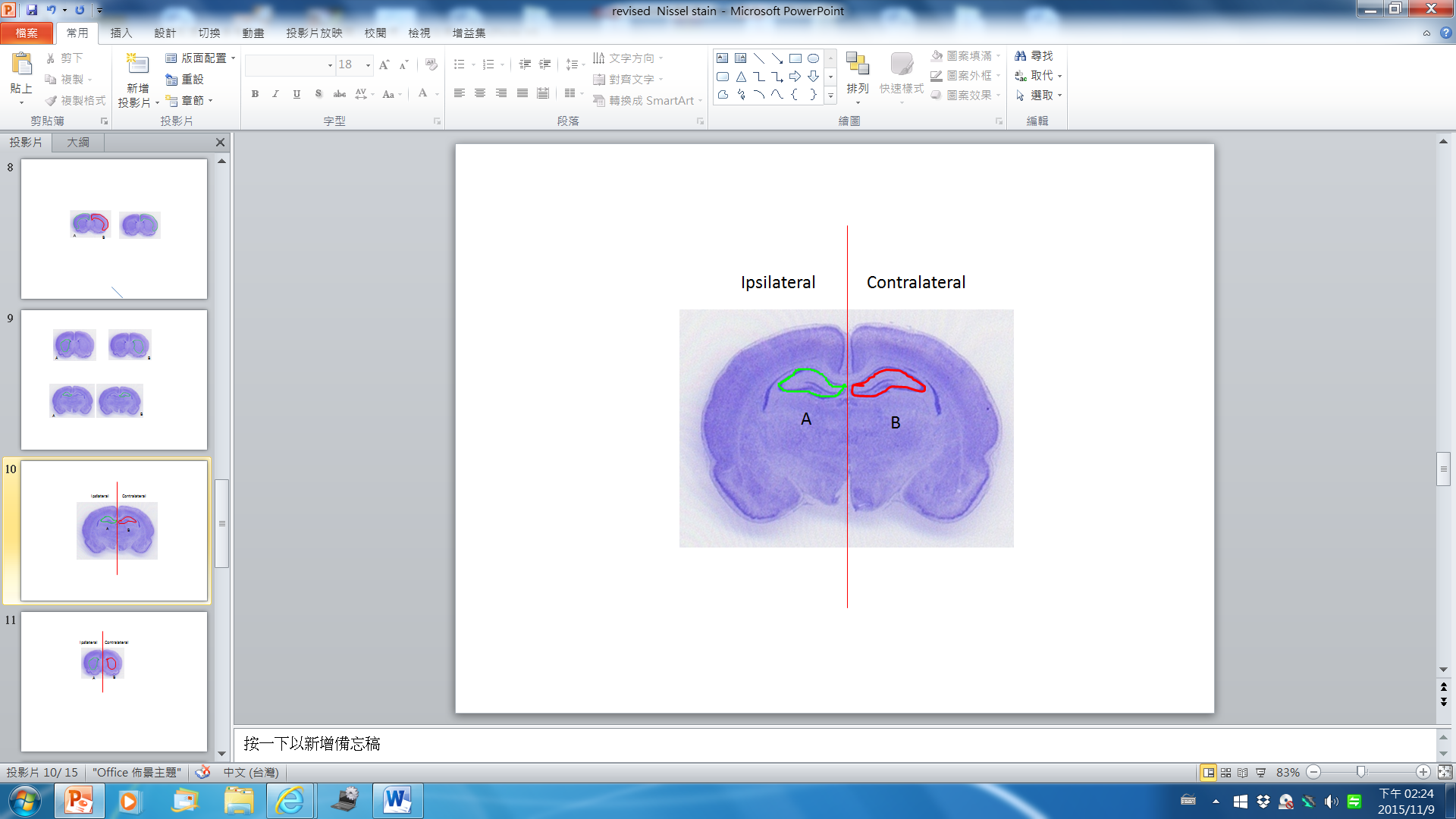


**Immunohistochemisty.**

For immunohistochemical staining, four coronal sections, two at the striatum level (0.26 mm and 0.92 mm posterior to the bregma) and two at the dorsal hippocampus level (3.14 mm and 4.16 mm posterior to the bregma), per rat were selected [18] according to the reference planes in a rat brain atlas [22] and assessed for each brain.

For the cell counting (ED1+ microglia, MPO+ neutrophils and CXCL5), MBP score, and IOD measurement (extravascular IgG, and GFAP) after immunohistochemistry, three visual fields within the medial, middle, and lateral areas in the white matter of hemisphere per section of the four selected sections per brain as described were analyzed. The data in four brain section were summed up to obtain the average data for each rat pup.

Quantification was performed at 400× magnification per visual field (0.0356 mm^2^) for CXCL5 signals and ED1(+) microglia, and at 200× magnification per visual field (0.145 mm^2^) for the MPO(+) neutrophils and extravascular IgG signals. Three visual fields within the medial, middle, and lateral areas in the white matter of each hemisphere per section and four sections per brain were analyzed and averaged. The mean IOD values in the white matter of the ipsilateral and contralateral hemispheres of each experimental group were compared with those of the control group to obtain the relative IOD ratios.

Three representative visual fields (medial, middle, and lateral areas) in the brain section chosen for immunohistochemical assessment are illustrated below


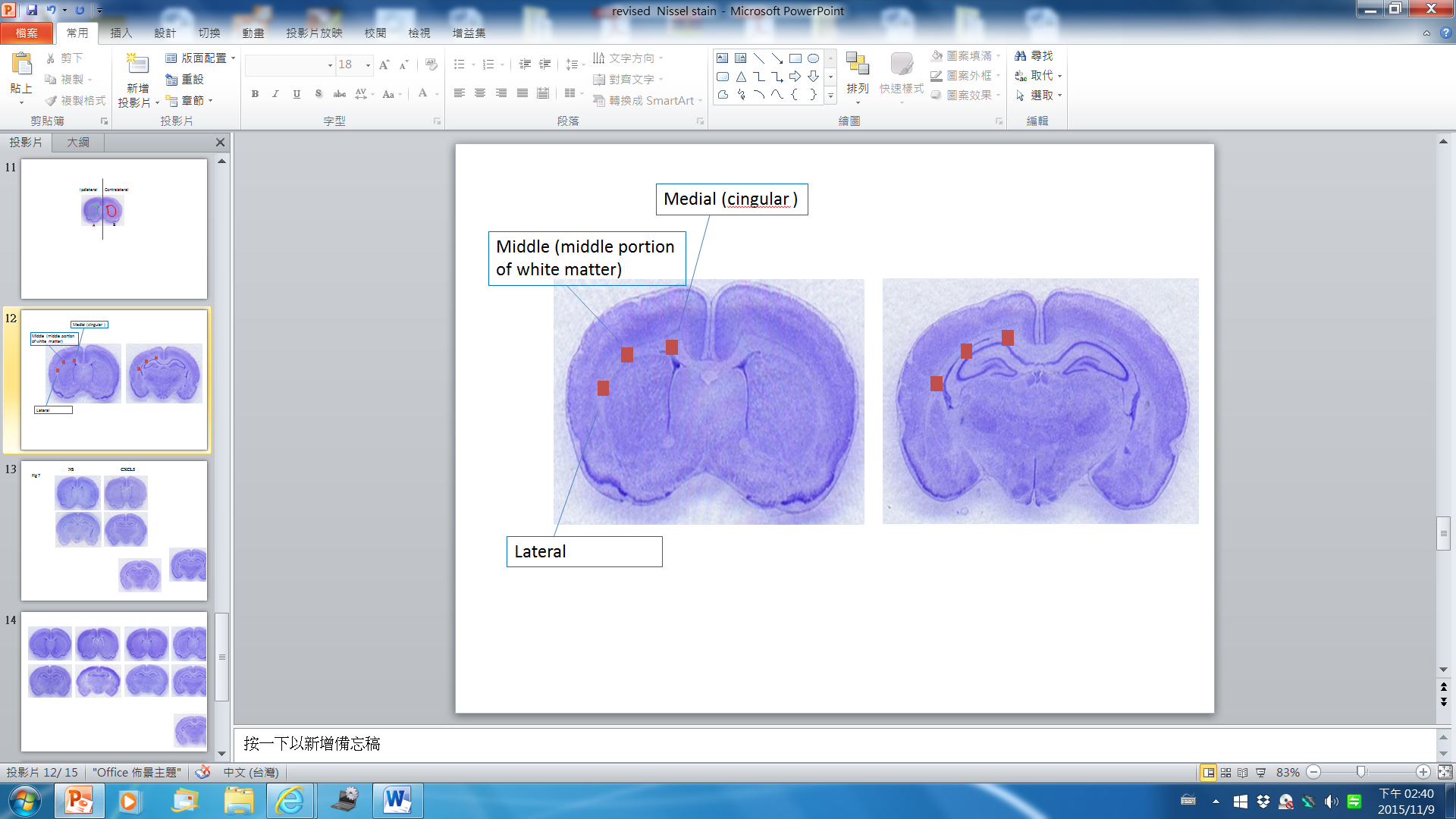


One way ANOVA was used to calculate the sample size required in each group using Gpower 3.1.9.2. For example, for the ventricle size ratio in each group in Figure 6A, the result shows the power was 90% for group size of 6 based on the ventricle size ratio in Figure 6A with the most conservative standard deviation of 4. So with a sample of 6 for each group in our study is adequate for analysis.


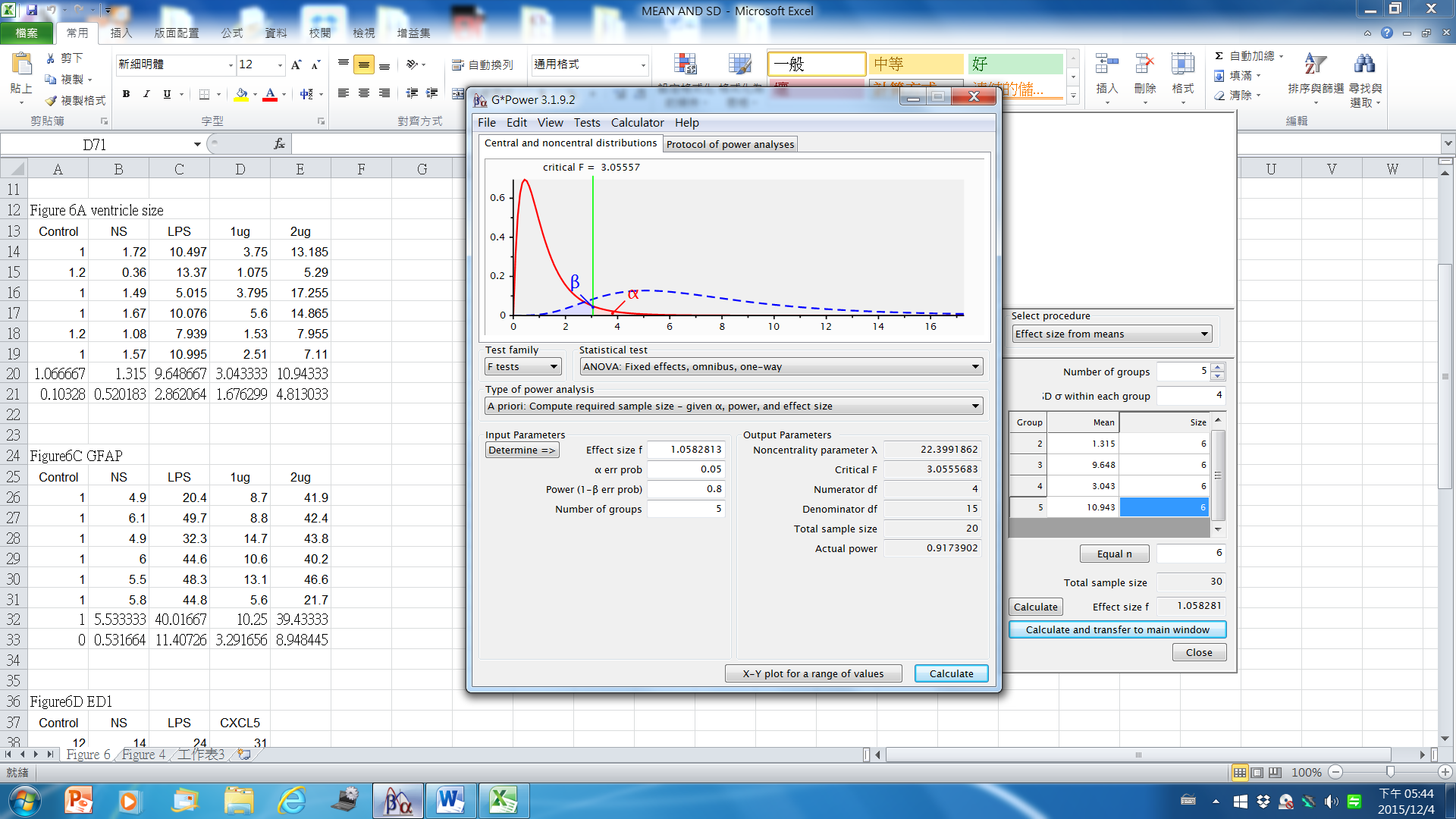


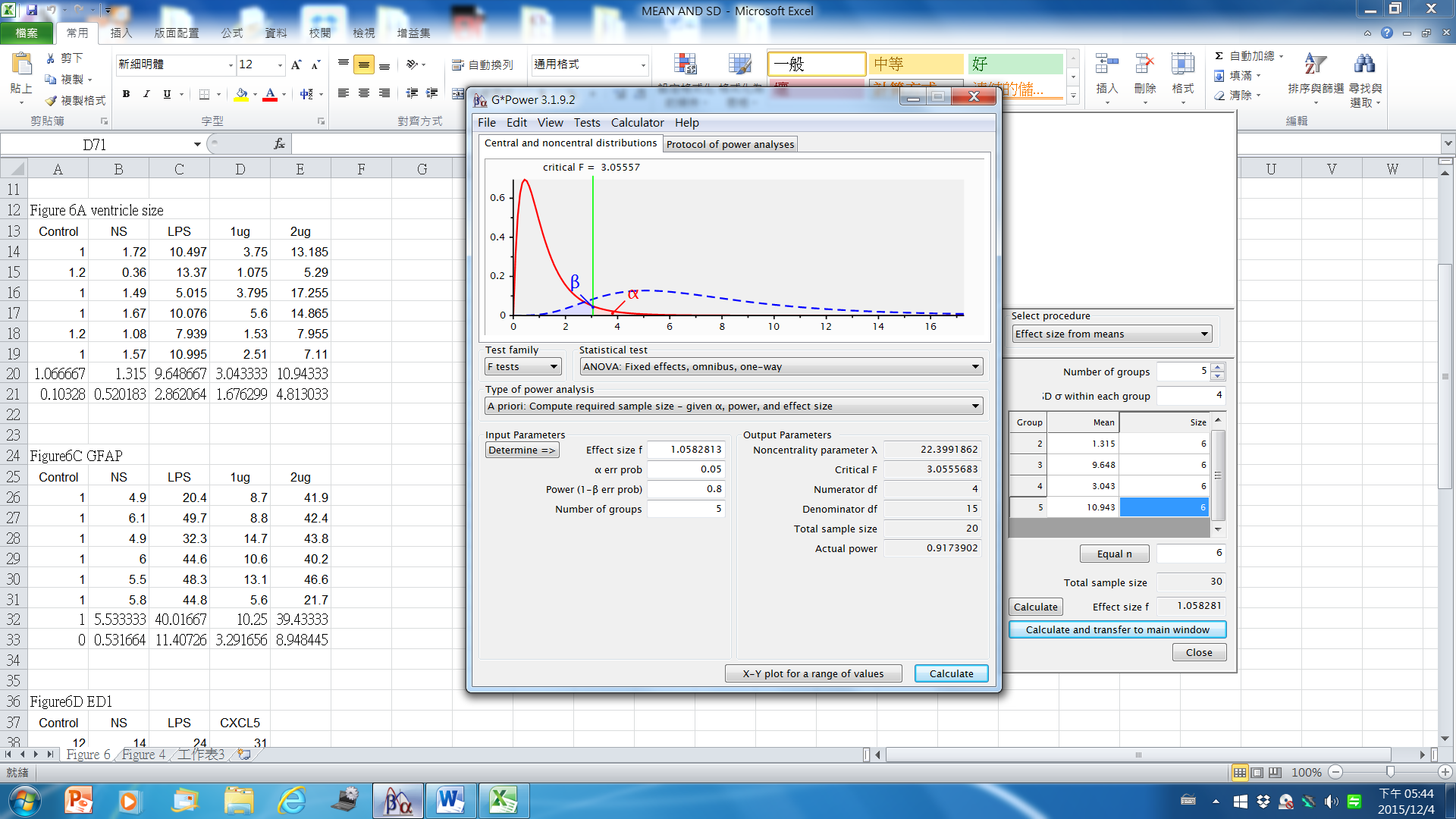


The MPO expression levels between the LPS+HI group and the LPS+HI group after SB225002 treatment.
